# Acute NMDA Receptor Antagonism Disrupts Synchronization of Action Potential Firing in Rat Prefrontal Cortex

**DOI:** 10.1371/journal.pone.0085842

**Published:** 2014-01-17

**Authors:** Leonardo A. Molina, Ivan Skelin, Aaron J. Gruber

**Affiliations:** Canadian Centre for Behavioural Neuroscience, Department of Neuroscience, University of Lethbridge, Lethbridge, Alberta, Canada; University of British Columbia, Canada

## Abstract

Antagonists of N-methyl-D-aspartate receptors (NMDAR) have psychotomimetic effects in humans and are used to model schizophrenia in animals. We used high-density electrophysiological recordings to assess the effects of acute systemic injection of an NMDAR antagonist (MK-801) on ensemble neural processing in the medial prefrontal cortex of freely moving rats. Although MK-801 increased neuron firing rates and the amplitude of gamma-frequency oscillations in field potentials, the synchronization of action potential firing decreased and spike trains became more Poisson-like. This disorganization of action potential firing following MK-801 administration is consistent with changes in simulated cortical networks as the functional connections among pyramidal neurons become less clustered. Such loss of functional heterogeneity of the cortical microcircuit may disrupt information processing dependent on spike timing or the activation of discrete cortical neural ensembles, and thereby contribute to hallucinations and other features of psychosis induced by NMDAR antagonists.

## Introduction

N-methyl-D-aspartate receptor (NMDAR) antagonists, such as ketamine and MK-801, have acute psychotomimetic effects in healthy subjects, including hallucinations, thought disorder, and impairments of working memory, pre-pulse inhibition, and attention [1], [2], [3], [4], [5]. NMDAR antagonists also precipitate acute psychotic episodes in schizophrenic patients [6]. Administration of NMDAR antagonists to rodents produces some behavioral phenotypes resembling aspects of schizophrenia symptomatology such as impairments of working memory, reduced pre-pulse inhibition, hyperlocomotion, stereotypies and increased social withdrawal [7], [8], [9], [10], and has thus been used as an animal model of acute psychosis related to this disease [11], [12]. Furthermore, the behavioral effects of NMDAR antagonists are suppressed by the concomitant application of clinically used antipsychotics [13], [14], suggesting that their mechanisms of action might be relevant to the pathophysiology of schizophrenia.

The prefrontal cortex (PFC) is one of the cortical regions most consistently implicated in the etiology of schizophrenia. This is supported by structural and functional neuroimaging [15], [16], [17], post-mortem studies of schizophrenic patients [18], [19], and the dependence of cognitive functions disrupted in schizophrenia, such as working memory and set-shifting, on the integrity of PFC (reviewed by [20]). Previous studies of NMDAR antagonist effects on neural activity in the rodent medial PFC have shown increased firing rates of putative pyramidal neurons and decreased firing rate of putative interneurons, leading to a state of cortical disinhibition [21]. Cortical disinhibition has been hypothesized to alter neural dynamics and impair neural information processing in schizophrenia by decreasing the signal content with respect to random ‘noise’ [12], [22], [23], [24], [25]. For instance, the random variation of brain signals measured by electroencephalography (EEG) is inversely related to cognitive functioning in schizophrenic patients [25]. The electrical activity of the brain has a complex frequency composition that changes with behavioral state. The amplitude of γ-band oscillations (30–90 Hz) correlates with working memory and other cognitive functions [26], [27]. Increased γ-band oscillations have been proposed to support cortico-cortical communication to facilitate the integration of spatially segregated neural ensembles and binding information from multiple sensory modalities into a coherent entity [28]. EEG oscillations in the γ-band are disrupted in schizophrenia [29] and by NMDAR antagonists [30]. The relationship between these changes in EEG power and the synchronization of action potentials remains unknown. Ongoing oscillations in the γ-band are drastically increased by NMDAR antagonists in humans [30] and rodents [31], [32]. Some neurons in the cortex tend to generate action potentials during certain phases of γ-oscillations [33], [34], suggesting that elevated γ-band power by NMDAR antagonists could increase synchronization of action potentials. On the other hand, the ability of these drugs to reduce firing of putative inhibitory interneurons [21] may disrupt interneuron-dependent synchronization of pyramidal neurons [35]. Here, we used high density electrophysiology to assess how rodent cortical dynamics are affected by acute NMDAR antagonism in order to elucidate possible functional deficits associated with cortical disinhibition.

## Materials and Methods

### Ethics Statement

All procedures for animal use were performed in accordance with the Canadian Council on Animal Care (CCAC) guidelines and were approved by the University of Lethbridge Animal Welfare Committee.

### Subjects and Surgical Procedure

**Figure 1:**
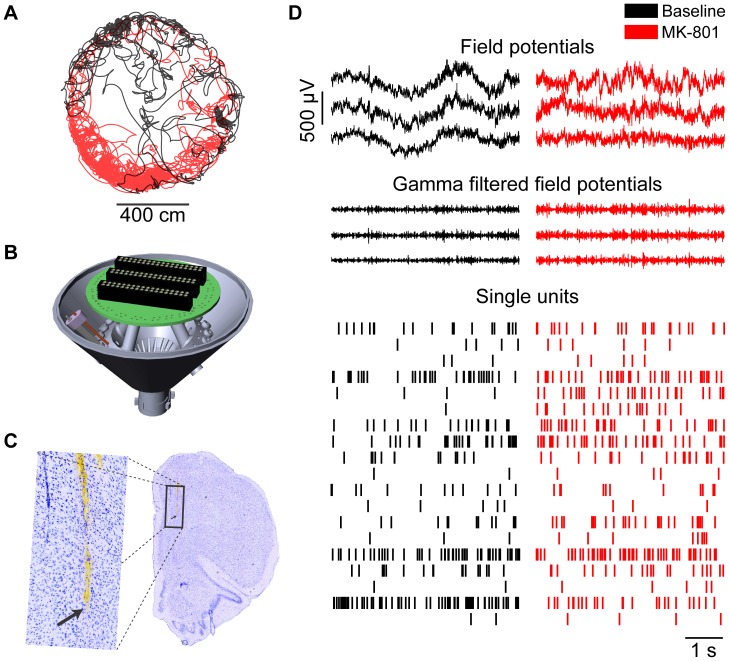
Behavioral and neural data. A) The position of subjects was monitored as they foraged in either a rectangular or round arena both before (black) and after administration of MK-801 (red) or vehicle (not shown). B) Computer rendering of drives used for high density electrophysiological recordings; only 1 out of 18 independently-drivable tetrodes is shown. C) Post mortem coronal brain section labeled with cresyl violet, showing electrode tracks in the medial prefrontal cortex. The arrow marks the bottom of the lateral track in the low magnification and high magnification (inset) photomicrographs. (D) Representative sample of neural signals recorded simultaneously during foraging. Only three of the FP signals (out of the 12–18 from each animal) and all of the single units recorded from the mPFC in one session are shown.

**Figure 2:**
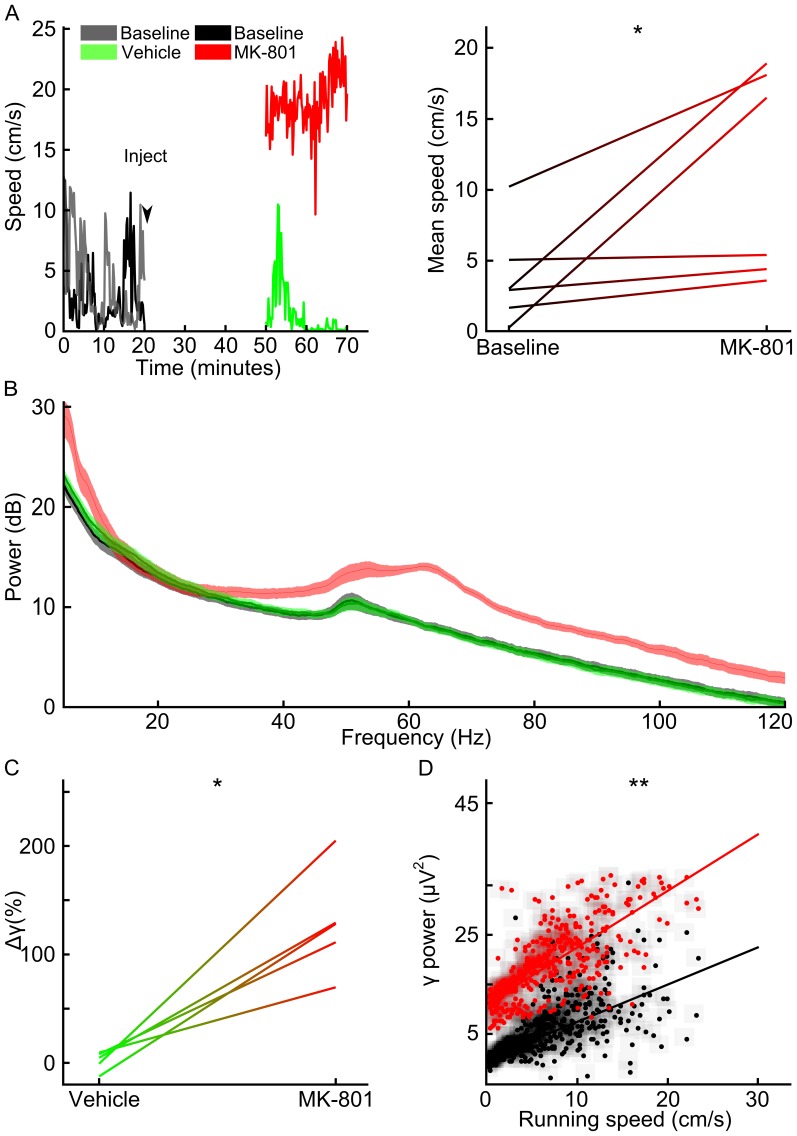
Relationship between running speed and FP power. A) Left: Running speed for a representative rat during one session in which vehicle was injected (green), and a different session in which MK-801 was injected (red). Right: Mean running speed before and after administration of MK-801 for the analyzed session from each subject. B) FP power before (black) and after injections. C) Change in γ-band FP power after injection of either vehicle or MK-801 for each subject. D) Plot of γ-band FP power vs. running speed before and after MK-801 injection for all subjects, showing that the increased γ-band power is independent of running speed. Data are shown as a density plot of Gaussian-smoothed points with an overlay of the linear fit. Single asterisk ‘*’ indicates statistical significance by paired t-test (p<0.05). Double asterisk ‘**’ indicates statistical significance by post-hoc test (p<0.05) following ANCOVA.

**Figure 3:**
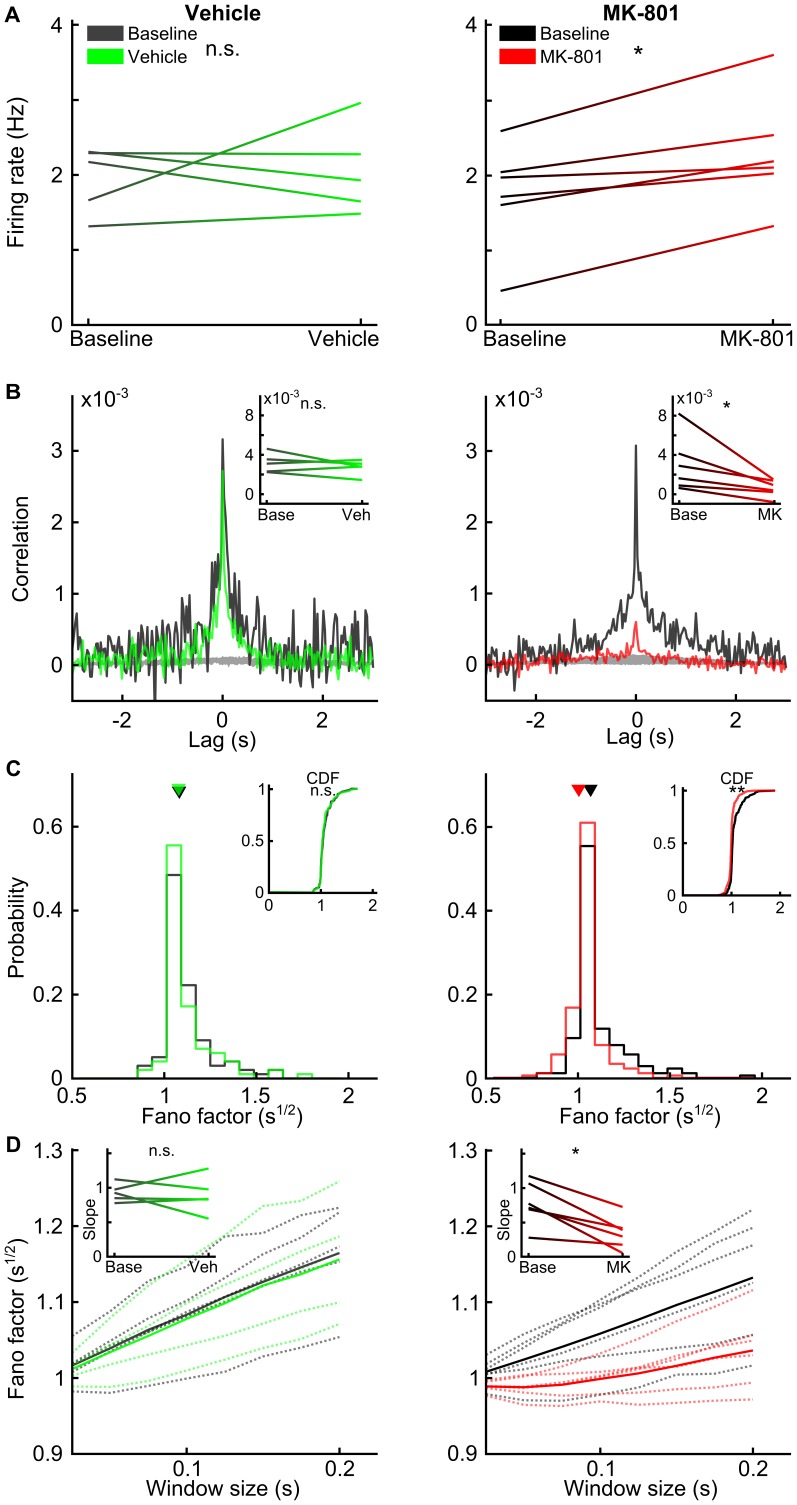
Effect of vehicle or MK-801 on action potential firing. A) Firing rate before and after injection of vehicle (left) or MK-801 (right) for each subject, showing elevated firing for drug but not vehicle. B) Mean cross-correlograms for all pairs of neurons with significant positive 0-lag correlations before (black) and after injections; surrogate data are displayed in gray. Inset shows the mean of 0-lag correlations before and after injection for each subject. C) Plot of Fano factor before (black) and after injection for all units. Cumulative probability distributions are shown in the inset. D) Plot of Fano factor for different time bin sizes averaged over all units for each animal before (black) and after injection. Inset shows regression coefficient for each line in the main plot paired before and after injection. Single asterisk ‘*’ indicates statistical significance by paired t-test (p<0.05). Double asterisks ‘**’ indicates statistical significance by the Kolmogorov-Smirnov test (p<0.05). Not significant indicated by ‘n.s.’

Six rats (5–7 months old, weighting 300–370 g at the time of the surgery; Brown-Norway or Brown-Norway Fischer hybrid) were individually housed in home cages in a 12 h reversed light-dark cycle room. Each rat was anesthetized with 1–2% isoflurane in oxygen at a flow rate of 1.2–2.5 L/minute and placed in a stereotaxic holder. Lidocaine (0.1 ml SC) was injected under the scalp prior to making an incision along the midline to access the skull. Craniotomies were made to allow recording electrodes to target the prelimbic regions of mPFC (3.2 mm anterior and 1.2 mm lateral (right hemisphere) to Bregma at 9° angle toward the midline; Figure 1C). Screws were implanted in the skull for structural support and to provide attachment of ground wires. A polymer-based adhesive (MedaBond) was applied to increase adhesion of dental acrylic to bone and the screws. Drives containing 12–18 independently-drivable tetrodes [36], [37] (Figure 1B) and 2–3 drivable reference electrodes were chronically implanted on the skull using dental acrylic. Prior to implantation, each electrode wire was individually gold-plated to achieve an impedance of 200–500 kΩ. Recording electrodes were lowered 0.5–1 mm ventral from brain surface immediately after the surgery and subsequently lowered up to 3–4 mm over the course of 2–3 weeks to reach the target region. Rats received post-surgical treatment for 2 days consisting of Metacam (1 mg/kg, SC, 2 times a day), Convenia (0.1 ml/kg, SC, once a day), and buprenorphine (0.03 mg/kg, SC, 1 time pre-surgery and 1 time during recovery). Post-mortem histology using cresyl violet was used to visualize electrode tracks so as to assess the positioning of the electrodes.

### Behavior, Electrophysiology and Data Pre-processing

Experiments were conducted in freely moving rats in a dimly lit room. Neuronal activity was recorded while animals explored a circular arena 1 m in diameter or square arena 0.6 m on each side. Foraging was encouraged by sparse distribution of chocolate sprinkles across arenas. A motorized slip ring (Neurotek) was used to allow subjects to rotate freely during electrophysiological recordings. A video tracking system (Neuralynx) was used to record the position of the rats during experiments (see Figure 1A).

Prior to the recording experiment, rats were brought daily to the laboratory and connected to the recording hardware in order to habituate them and to monitor electrical signals while advancing the tetrodes. Once the tetrodes were estimated to be at the proper depth and neurons were ‘tuned in’ on as many channels as possible, experimental data were collected. Rats were connected to the recording hardware and allowed to explore a familiar open field for 30 minutes. They were then administered either vehicle (0.2 ml/kg of 0.9% NaCl) or MK-801 (0.1–0.2 mg/kg in 0.2 ml/kg of vehicle) in the intraperitoneal space, and returned shortly after to the arena for another 50 minutes. Only one session was recorded each day, and we alternated between drug and vehicle injections on consecutive recording sessions. Tetrodes with excellent signal (multiple neurons with good clustering in feature space) remained at the same location across consecutive sessions of drug and vehicle injection. Other tetrodes were individually advanced 20–80 microns under auditory and visual feedback of detected waveforms in attempt to maximize the number of neurons recorded across all tetrodes. This sequence was repeated 1–5 times for each subject, and the one drug session and one control session with the most neurons recorded from each subject was used for analysis. A vehicle session is missing from one subject due to hardware malfunction. We did not assess any cumulative or long-term effects of repeated drug administration, but rather investigated acute effects of NMDAR antagonism as compared to those of vehicle administration. We analyzed neuronal recording data from the last 20 minutes of the post-injection period so as to allow the drug to take effect, and compared this to the 20 minute period prior to injection. Signals from intracortical electrodes were first amplified by a unity-gain headstage (Neuralynx) to reduce noise susceptibility during signal transmission to the data acquisition system (Neuralynx Cheetah) via a multi-wire cable and slip ring mounted on the ceiling. Input signals were amplified 1250 times, digitized at 32000 Hz, and band-pass filtered differently for field potentials and spikes before saving to a computer hard drive. For the field potentials (FP), the signal was band-pass filtered between 1–1000 Hz and downsampled to 2000 Hz. For spike data, the signal was filtered between 600–6000 Hz and 2 ms-long samples were recorded whenever the amplitude of the signal crossed a pre-defined threshold between 35–50µV.

### Data Analysis

Data were further processed for off-line analysis using SPSS (IBM), Matlab (Mathworks) and open-source Matlab toolboxes for computing spectra (Chronux toolbox; [38]). Field potential data were first band pass filtered (4–200 Hz) using a zero-phase digital filter. Power spectra of field potentials were computed by the fast Fourier transform algorithm using a standard multi-taper windowing method on 100 non-overlapping segments (3 seconds) randomly sampled from the filtered field potential for each epoch (pre-injection or post-injection). Confidence intervals of the power spectra were computed by jackknife error estimates [38] for individual sessions. For group data, the spectra error bars are the standard error of the mean. To investigate the effect elicited in the γ-band, analyses were limited to the frequency range 30–90 Hz by filtering the originally acquired data with a different zero-phase digital filter. The relative change in γ-band power (ratio of the power in the post-injection period to power in the pre-injection period) was used to account for variability in field potential amplitude between recording sessions when displaying population averages. To assess whether γ-band power covaried with running speed, we used analysis of covariance (ANCOVA) on averaged γ and speed data binned over 750 ms. Two-way analysis of variance (ANOVA) with repeated measures was used for other multi-factor statistical comparisons.

We analyzed the relationship between the phases of γ-band FP recorded simultaneously on separate electrodes using a recently developed method for detecting intermittent phase locking [39]. We first extracted the instantaneous phase of each γ-band FP signal with a Hilbert transform. We then examined the phase relationship of every pairwise combination of simultaneously recorded FP signals. We chose one of the pair to be a reference signal, and assessed the phase of the non-reference signal when the phase of the reference signal was 0 radians. The phase difference indicated which signal was leading in that cycle. We then determined the number of consecutive cycles with consistent phase relationship. To determine if such *phase slip* depended on the amplitude of γ-oscillations, we repeated the analysis using data binned over 2 s. Average γ-band power and the length of sequences with consistent phase relationship in these windows were used to compute the phase slip ratio, which is the ratio of sequence counts: (number of length = 1 sequences)/(number of length>1 sequences).

Action potential waveforms were classified as belonging to individual neurons using computer algorithms and subsequent manual verification. First, a clustering algorithm (KlustaKwik; [40]) was used to group spike waveforms into clusters based on numerous waveform features. A modified version of an open-source software (MClust, [41]) was then used to manually merge, split, or delete clusters based on standard assumptions about the characteristics of well-isolated clusters such as stable amplitude, few (<0.5%) spikes with inter-spike-interval shorter than standard refractory period (2 ms), and unimodal distributions in feature space [42]. A firing rate criterion (0.15< firing rate <8 Hz) was then used in attempt to restrict neurons in the analysis to putative pyramidal neurons. Cross-correlations provided a measure of synchronization between neurons: Single-unit activity was first transformed into a 30 ms binned representation to resolve short-range interactions associated with γ-frequencies (>30 Hz). Then a z-score transform was applied to normalize firing rates so that resultant computations were not strongly influenced by a few neurons with high firing rates. Cross-correlations were computed by:
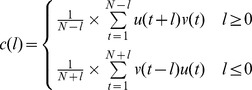
which reduces to the following equation for 0-lag correlation:



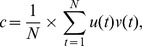
where *N* is the size of binned spiking vectors *u* and *v*, *l* is the offset between them, and *t* is the time index of the spikes. Confidence intervals for these correlations were estimated by performing multiple correlations with surrogate data generated by a shuffling procedure. We first shuffled the inter-spike intervals of one of the two spike trains from every pair, and then computed their cross-correlation. This procedure was repeated 100 times for every unique pair of neurons. To assess changes in correlation following injections, we computed the average 0-lag correlation for all pairs of units that had significant 0-lag correlation either before or after injection, as determined by a correlation value greater than the 2 standard deviation confidence interval computed by the shuffling procedure.

To investigate changes in spike firing and gamma oscillations, we calculated cross-correlations between multi-unit activity and field potentials in the gamma range. Spiking data from each tetrode were cleaned from noise and added together to form multi-unit activity, then transformed into a binned representation with the same temporal resolution as the FP signal. Confidence intervals were estimated by the cross-correlations of multi-unit by surrogate data created by shuffling the phases of field potential.

We also characterized the time-variability of spikes by computing their Fano factor. First, spikes were randomly eliminated from the post-injection periods so as to match the firing rate in the pre-injection periods. Then, spikes were divided into time windows of a fixed size. The Fano factor was then computed as the ratio of the variance to mean of the spike counts within those windows.

## Results

We used recording drives with independently moveable tetrodes to record neural activity from the prelimbic region of mPFC as rats foraged in an arena (Figure 1). Administration of MK-801 (0.1–0.2 mg/kg) caused a significantly increased locomotion (Figure 2 A; one-tailed t-test; t(5) = 2.45, p<0.03). Administration of MK-801 significantly increased γ-band power in the field potential (FP) signals recorded within the mPFC (Figure 2 B-C; two-tailed t-test; t(4) = 5.31, p<0.006), consistent with previous reports [5], [43]. The spectral features of γ-band FP in the frontal cortex correlate with behavioral speed in rats [44]. The increased locomotion evoked by MK-801 thus presents a possible confound for increased γ-band power. We therefore used analysis of covariance between γ-band power and running speed, with drug as a categorical predictor. This analysis shows that γ-band power increases with running speed both before and after MK-801 administration, and that the drug significantly increases γ-band power across all running speeds (Figure 2 D; ANCOVA; F(2,766) = 931.17; p<0.0001). Thus, hyperlocomotion partially confounds the increase in γ-band power because rats spend more time running at higher speeds following drug administration, but a significant effect of drug remains after correcting for such locomotion effects, consistent with other reports [24].

A total of 271 neurons met the criterion for inclusion in the analysis. We first investigated the effects of MK-801 on the firing of action potentials (Figure 3). The firing rate of neurons increased following MK-801 (two tailed paired t-test; t(5) = 4.20, p<0.009), but not after vehicle (paired t-test; t(4) = 0.34, p>0.75) administration, consistent with previous reports [21]. To our knowledge the correlation among action potentials from different units has not been previously reported in this drug model. We found that the proportion of neuron pairs with significant positive correlation was not altered by either vehicle (McNemar test; χ^2^ = 1.66, p>0.20) or MK-801 administration (McNemar test; χ^2^ = 0.99, p>0.32). The magnitudes of pair wise spike-spike correlations, however, were significantly reduced by MK-801 (paired t-test of the session mean of significant 0-lag correlations; t(5) = 2.69, p<0.05) but not vehicle (paired t-test of the session mean of significant 0-lag correlations; t(4) = 1.03, p>0.36). Even though we focused our study in the 30 ms range, a significant reduction of 0-lag correlation was consistently found across a range of bin sizes (10–100 ms) after injection of MK-801 (see Figure S1). The variability of spike timing from individual neurons is also informative of dynamical processing. One metric for characterizing such variability is called the Fano factor, which is the ratio of the variance to the mean rate of action potential firing. The Fano factor has a value of 1 for Poisson processes, in which the likelihood of an event is independent of past events. Larger Fano factor values indicate increased variability, as would be expected for neurons that transiently modulate their mean firing rate. We found that MK-801 administration significantly shifts the distribution of Fano factors for all units toward lower values (Kolmogorov-Smirnov test; D(173) = 0.36, p<0.0001), whereas vehicle administration did not evoke a shift (Kolmogorov-Smirnov test; D(98) = 0.11, p>0.55). We further found that this effect was not dependent on the time window of analysis; the Fano factor was consistently reduced across subjects after MK-801 administration (paired t-test of slopes from each subject; t(5) = 4.59, p<0.006) but not after vehicle administration (Figure 3 D; paired t-test of slopes; t(4) = 0.31, p>0.77). Together, these data indicate that MK-801 alters the neural processing in mPFC such that neurons generate action potentials more independently from one another.

**Figure 4:**
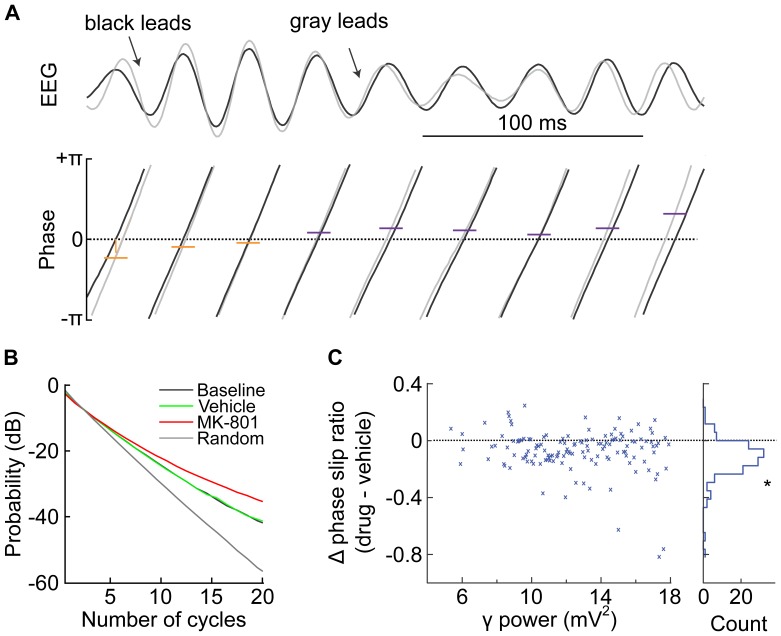
Analysis of FP phase relationship among electrodes. A) Sample trace of γ-band FP recorded simultaneously from two different electrodes in mPFC. The black trace leads the gray trace for the first 3 cycles, and then the phase relationship inverts such that the gray trace leads. B) Probability of the number of consecutive cycles of constant phase relationship of simultaneously recorded γ-band FP before (black) and after injections. Data are aggregated from all subjects. MK-801 administration leads to a higher probability of longer sequences of constant phase relationship. All conditions show structure different from a random condition in which the phase relationships are shuffled (gray). C) Plot of the difference in phase slip ratio as a function of γ-band power, showing a reduction across the range of γ-band power following MK-801 administration. A histogram of these values (right side) shows a distribution with a mean different from 0 (t-test, p<0.0001).

The drug-induced reduction in firing synchronization despite increased γ-band FP power could potentially arise from reduced correlation between the FP and unit firing, or from reduced synchronization among FP recorded on different electrodes. We found, however, that the 0-lag correlation between pairs of γ-band FP was not affected by MK-801 administration (paired t-test; t(5) = −0.47, p>0.66), and that the 0-lag correlation between γ-band FP and multi-unit activity (summed activity from all units) was not affected by MK-801 (paired t-test; t(5) = 1.95, p>0.11). This suggests that aggregate neural activity is not grossly affected by drug administration. These methods, however, are not sensitive to some types of interactions, such as intermittent phase locking [39]. We thus next used a method to assess how MK-801 affected the phase relationship among pairs of γ-band FP recorded simultaneously from different electrodes (Figure 4). We first determined which signal was leading (advanced phase) on each cycle of γ-oscillation. We then computed the number of consecutive cycles between each inversion of the phase relationship. We found that vehicle administration did not affect this measure (compare black and green lines in Figure 4 B), but that MK-801 administration caused a shift towards longer sequences of consistent phase relationship. The reduced amplitude of γ-band oscillations in the vehicle condition as compared to the MK-801 condition could produce an artifact in this metric by contributing more randomness to the estimate of instantaneous phase in the vehicle case. To control for such an artifact, we computed the phase slip ratio for bins of γ-band power in both vehicle and MK-801 conditions. The difference of this ratio between conditions was consistently negative for the range of γ-band power. The distribution of these differences was significantly different from zero (paired t-test; t(5) = −5.65, p<0.0001), indicating that the phase relationship is more stable following MK-801 administration as compared to vehicle regardless of γ-band power. Together, these analyses indicate that the reduced spike-spike correlations are not due to gross changes in the relationship between field potentials and neural firing as measured by multi-unit activity, nor due to increased variation in γ-band oscillations across electrodes.

## Discussion

We found that acute administration of the NMDAR antagonist MK-801 increased the power and phase relationship of γ-band FP in the mPFC, but decreased neuronal synchronization, as reflected by the decreased mean pair-wise correlations among unit firing. Moreover, spike trains from individual units were more random following drug administration, as indicated by a lower Fano factor (ratio of the variance to the mean of firing rate). Thus, MK-801 evokes more disorganized spiking activity despite the increase in γ-band FP signal.

These dynamical changes could result from a state of disinhibition, in which insufficient inhibition leads to excessive excitation. Cortical inhibitory interneurons are more efficiently recruited by NMDAR-mediated excitatory inputs than are pyramidal neurons [45], presumably due to the higher expression levels of the NMDA NR2A subunit than pyramidal neurons [46]. This subunit is more responsive to NMDAR antagonists than the type more commonly found on pyramidal neurons [47]. This renders interneuron spiking more sensitive to the direct suppressive effects of mild NMDAR antagonism [21]. MK-801 may thus corrupt the interneuron-mediated temporal regulation of pyramidal neuron firing, and consequently information processing utilizing temporal coding [35], [48].

The more disorganized action potential firing induced by NMDAR antagonism may reflect important changes in the functional organization of cortical processing. Recent computational studies have shown that the Fano factor of neuronal firing rates in simulated cortical networks is inversely related to the clustering of unit connectivity [49]. Networks with high clustering form multiple groups of neurons with many strong connections between units belonging to the same group, and fewer connections with units in other groups. This type of connectivity leads to so-called ‘small-world networks’ (SWN) that have been studied extensively in many domains of science [50], [51]. Simulated cortical networks show that the Fano factor and pair-wise correlations among neurons are reduced as clustering is reduced [49]. Thus, the reduction in these metrics following MK-801 administration reported here suggests that the disinhibited cortex may become functionally less clustered. This would likely cause neural firing to be less specific. Indeed, disinhibition induced by the GABA-A receptor antagonist bicuculline broadens the directional tuning of neurons in primate PFC [52], [53]. It is conceivable that less selective tuning of mPFC neurons receiving sensory information [54] could contribute to aberrant association of information from different sensory modalities, with possible implications for hallucinatory phenomena.

The present findings suggest that MK-801 administration could temporarily augment the mPFC functional connectome, making it less like a SWN. This may have important implications for information processing. Theoretical studies have shown that the SWN architecture is optimal for both specialized information processing at the modular level, as well as the integration of information across distant brain structures [55]. Interestingly, scalp-recorded FP and fMRI studies of schizophrenic patients show disruption of SWN-like connectivity at the macroscopic level [56], [57]. Episodes of high synchronization among subsets of units is one of the hallmarks of SWN [51]. The decreased synchrony of spiking after MK-801 administration in the present study suggests the loss of SWN-like properties at the mPFC microcircuit level. Furthermore, the clustering coefficient, defined as the ratio of the mean within-cluster and network-level connection probabilities, is another feature of SWN that is reduced in the brains of schizophrenic patients [56], [58]. These data suggest that the NMDAR antagonist model of acute psychosis associated with the early phases of schizophrenia [12] produces augmentation of mPFC functional connectivity at the microcircuit level that may mimic aberrant macroscopic function in schizophrenia. In both cases, the cortex appears to function as a less-clustered network.

The administration of MK-801 in the present study increased γ-band FP power in the rat mPFC. Although increased locomotion is a confounding factor, our ANCOVA analysis reveals a drug effect independent of movement. This is in accordance with a number of studies showing that the systemic administration of NMDAR antagonists increases the power of γ-band FP power in a variety of structures, both in anaesthetized and behaving rats, as well as slice preparations [21], [24], [32], [59], [60]. Some groups have reported an increase in γ-band EEG power in schizophrenia [15], [61], [62]. Other groups report a decrease in γ-band EEG, and suggest this is functional evidence of reduced neural synchronization [25], [63], [64], [65], [66]. This argument rests on the assumption that scalp-recorded EEG power reflects synchrony among neural firing of action potentials. The dissociation of changes in γ-band FP power and neural synchronization in the present study indicates that this assumption does not hold for NMDAR-induced increases in γ-band field potential power, and raises the possibility that it may not be valid in other circumstances as well. The inability of inputs oscillating in the γ-range to synchronize the local neuronal population in our data set is in accordance with the disconnection hypothesis of schizophrenia, which predicts impaired functional integration between the distributed networks that support higher cognitive functions [67].

Gamma oscillations in the cortex and hippocampus emerge as a network property dependent on the interaction of GABAergic interneurons with excitatory pyramidal neurons, and canonically increase synchronization of pyramidal neuron firing [68], [69], [70]. These oscillations are supported by AMPARs, and γ-band amplitude increases as the relative contribution of the slower NMDARs to the post-synaptic current decreases [70]. However, the traditional gamma rhythm may be comprised of multiple components with distinct relationships to neural processing [71]. Interestingly, the amplitude of higher frequency components of the gamma rhythm are correlated with firing rate in monkey visual cortex, whereas the lower frequency components are not [72]. This suggests that the lower frequency components constitute a brain rhythm independent of firing rate, and may explain why we found reduced synchronization among pyramidal neuron firing despite an increase in gamma amplitude. Subthreshold membrane voltage fluctuations are considered major contributors to the field potential [73], but they are not necessarily correlated with the spiking activity [74]. Coupling between the spiking and γ-band FP power seems to depend on the correlation of individual neuronal spiking [75]. Another demonstration of γ-band FP power decoupling from neuronal synchrony was obtained using optical stimulation of light-sensitive pyramidal neurons in mouse barrel cortex, which had no significant effect on γ-band power despite a substantial increase in firing synchrony [76]. These data indicate a complex relationship between action potential generation and field potential oscillations, which may be disrupted in disinhibited states.

## Conclusion

Our results support the hypothesis that cortical disinhibition induces less synchronization of spike firing, at least during some behaviors. This may correspond to ‘noisy’ information coding hypothesized to be present in schizophrenia [12], [22], [23], [24], [25]. The decreased synchrony and more random spike trains suggest that NMDAR antagonism renders the mPFC functional connectome less like a small-world network, in accordance with macroscopic level neuroimaging findings in schizophrenic patients [56], [58]. Investigating commonalities in aberrant neural synchronization and altered functional connectivity by other drugs that can induce hallucinations and delusions, such as amphetamines and serotonin-increasing compounds, may shed further light on what aspects of neural signaling are tied to psychosis.

## Supporting Information

Figure S1 **Effect of bin size on 0-lag spike-spike correlations.** Each data point corresponds to the 0-lag correlation values averaged over all pairs of neurons for each animal. The correlation is significantly reduced by MK-801 administration for bin sizes within the range 10–100 ms (compare black and red crosses) as indicated by the paired t-test on the session mean for which the p-values were smaller than 0.004 (asterisks connected by the red line). Vehicle administration did not significantly reduce the correlation consistently in this range (compare black and green circles) for which the p-values were larger than 0.1 (green asterisks) for all but one bin size.
(TIF)
