# Correction: Acute NMDA Receptor Antagonism Disrupts Synchronization of Action Potential Firing in Rat Prefrontal Cortex

**DOI:** 10.1371/journal.pone.0104110

**Published:** 2014-07-25

**Authors:** The PLOS ONE Staff

There are errors in Figures 1 and 4. Please see the corrected figures below.

**Figure 1:**
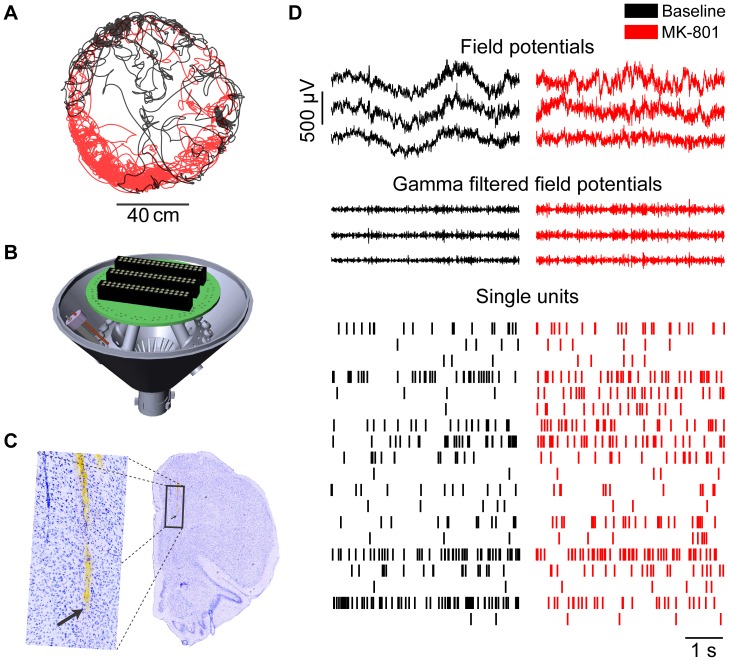
Behavioral and neural data. A) The position of subjects was monitored as they foraged in either a rectangular or round arena both before (black) and after administration of MK-801 (red) or vehicle (not shown). B) Computer rendering of drives used for high density electrophysiological recordings; only 1 out of 18 independently-drivable tetrodes is shown. C) Post mortem coronal brain section labeled with cresyl violet, showing electrode tracks in the medial prefrontal cortex. The arrow marks the bottom of the lateral track in the low magnification and high magnification (inset) photomicrographs. (D) Representative sample of neural signals recorded simultaneously during foraging. Only three of the FP signals (out of the 12–18 from each animal) and all of the single units recorded from the mPFC in one session are shown.

**Figure 4:**
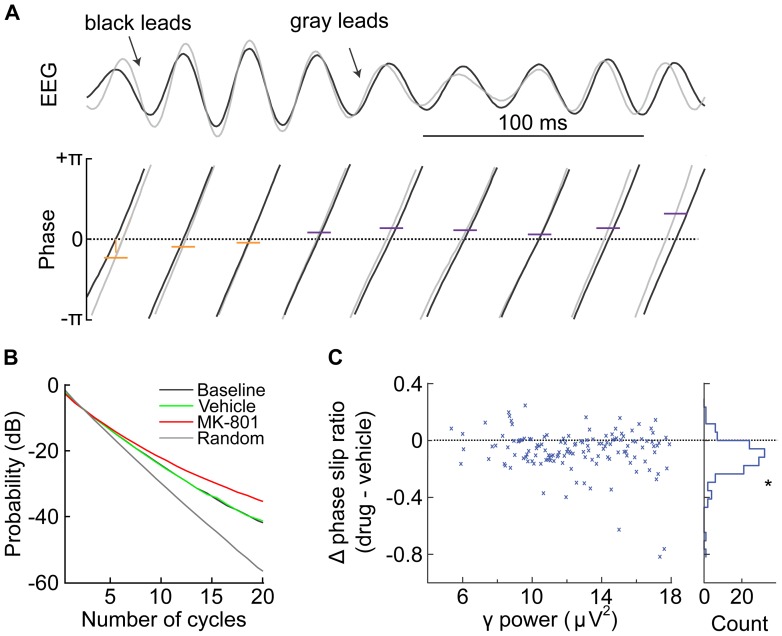
Analysis of FP phase relationship among electrodes. A) Sample trace of γ-band FP recorded simultaneously from two different electrodes in mPFC. The black trace leads the gray trace for the first 3 cycles, and then the phase relationship inverts such that the gray trace leads. B) Probability of the number of consecutive cycles of constant phase relationship of simultaneously recorded γ-band FP before (black) and after injections. Data are aggregated from all subjects. MK-801 administration leads to a higher probability of longer sequences of constant phase relationship. All conditions show structure different from a random condition in which the phase relationships are shuffled (gray). C) Plot of the difference in phase slip ratio as a function of γ-band power, showing a reduction across the range of γ-band power following MK-801 administration. A histogram of these values (right side) shows a distribution with a mean different from 0 (t-test, p<0.0001).
