# Supplementary figures and images for: Acute NMDA Receptor Antagonism Disrupts Synchronization of Action Potential Firing in Rat Prefrontal Cortex
Source: PLoS One. 2014 Jan 17;9(1):e85842. doi: 10.1371/journal.pone.0085842 (PMC3895008; doi:10.1371/journal.pone.0085842)

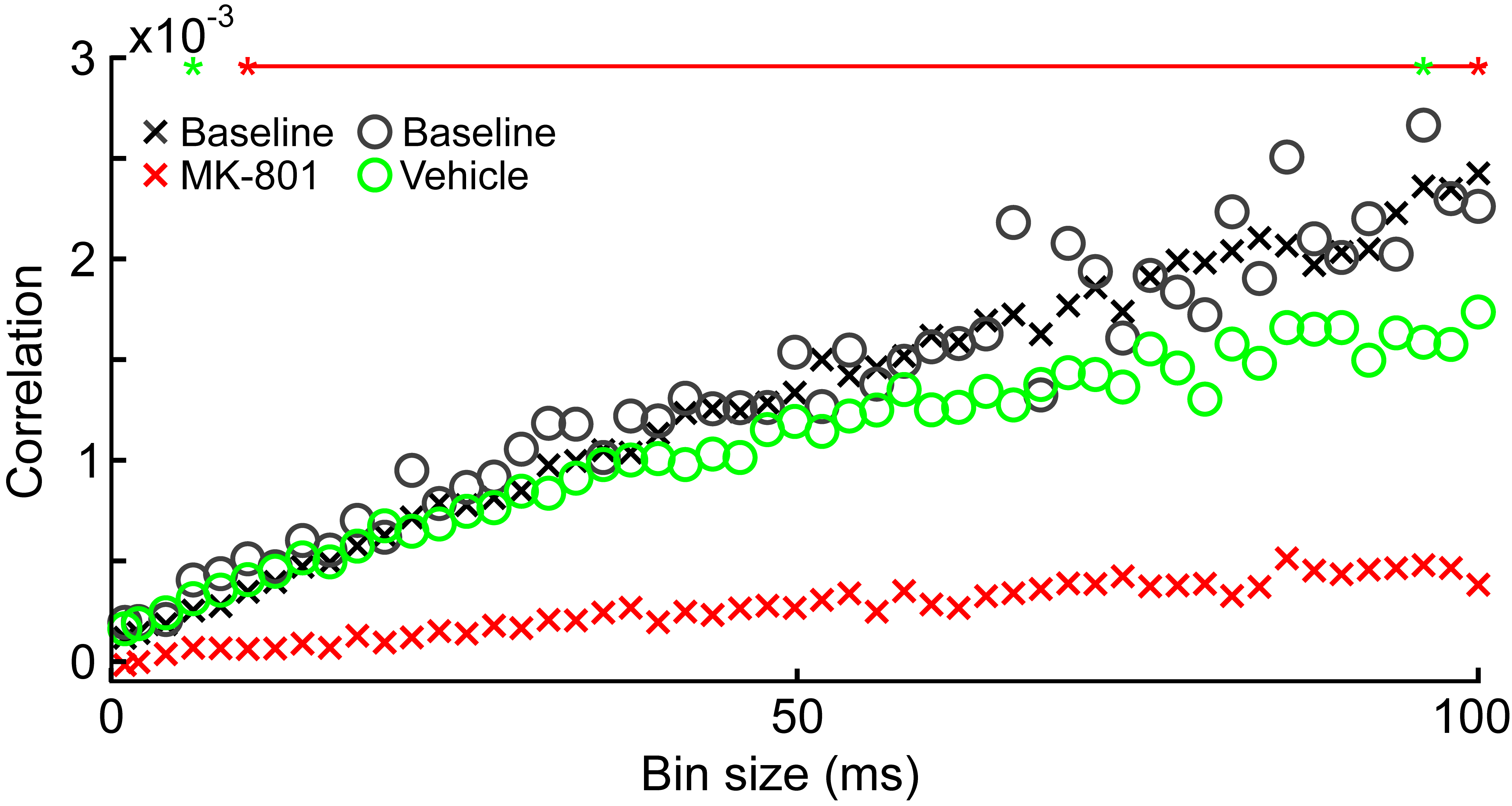

Supplement: Figure S1 — Effect of bin size on 0-lag spike-spike correlations. Each data point corresponds to the 0-lag correlation values averaged over all pairs of neurons for each animal. The correlation is significantly reduced by MK-801 administration for bin sizes within the range 10–100 ms (compare black and red crosses) as indicated by the paired t-test on the session mean for which the p-values were smaller than 0.004 (asterisks connected by the red line). Vehicle administration did not significantly reduce the correlation consistently in this range (compare black and green circles) for which the p-values were larger than 0.1 (green asterisks) for all but one bin size. (TIF) [file pone.0085842.s001.tif]
